# *Bacillus amyloliquefaciens* G02 enhances selenium uptake in lettuce (*Lactuca sativa*) by improving soil selenium availability and rhizosphere microbiome regulation

**DOI:** 10.3389/fmicb.2025.1656037

**Published:** 2025-09-26

**Authors:** Bingzhi Huang, Bei Li, Guofei Pan, Danni Huang, Guoming Yang, Yanmin Ma, Fengshuo Ya, Mingliang Rao, Yanyan Wei

**Affiliations:** ^1^State Key Laboratory for Conservation and Utilization of Subtropical Agri–bioresources, Guangxi Key Laboratory for Agro-Environment and Agro-Products Safety, National Demonstration Center for Experimental Plant Science Education, College of Agriculture, Guangxi University, Nanning, China; ^2^Customs Technical Center, Nanning, Guangxi, China; ^3^Ningming Jinming International Trade Development Co., Ltd., Nanning, Guangxi, China

**Keywords:** lettuce, selenium, plant growth-promoting bacteria, rhizosphere microbial, metabolomics

## Abstract

Selenium (Se) is an essential trace element for human, but its low availability in soils results in its inadequacy in edible crops, thereby limiting its human intake. However, the role of the plant growth-promoting bacteria in soil Se availability and the pathways involved in biofortification in edible plants remain poorly understood. In this study, a Se-tolerant, plant growth-promoting bacterium, *Bacillus amyloliquefaciens* strain G02, which activates Se was isolated from the soils in Se-rich fields in Guangxi, China. We employed soil microcosm and potted experiments, along with metabolomics and 16S rRNA sequencing, to investigate how strain G02 incubation promotes elemental Se (0) solubilization, soil Se activation, and Se enrichment in lettuce. The strain G02 exhibited high phosphate solubilization (87.36 mg/L), IAA production (8.35 mg/L), as well as siderophore and ACC deaminase activities. Strain G02 is capable of dissolved Se(0) and Se minerals, increased pH, and secreted metabolites enhancing Se solubility. Soil microcosm experiments showed that the incubation of strain G02 increased available Se forms [soluble selenium (SOL-Se) and exchangeable selenium (EXC-Se)] in soil. Moreover, potted experiments revealed that the incubation of strain G02 increased biomass, Se concentration in lettuce, soil enzyme activities, beneficial microbial abundance and the native bacterial taxa. The strain G02 enhances soil Se availability through metabolites secretion, Se solubilization, and rhizosphere microbial regulation, improving ability of lettuce to absorb and transport Se. This study provides novel insights into the microbially mediated Se biofortification.

## 1 Introduction

Selenium (Se) is an essential nutrient for human health, supplied through dietary intake to maintain normal physiological functions (Su et al., 2024). A daily intake of 40–400 μg of Se is recommended for adults, but intake levels below 40 μg lead to its deficiency, which subsequently causes various health issues, including hypertension, hyperlipidemia, hyperglycemia, cardiovascular diseases, and cancers (Brigelius-Flohé, 2018; Huang et al., 2024). Studies have shown that in plants, Se primarily exists in the form of organic Se [such as selenomethionine(SeMet) and selenocysteine(SeCys)], secondarily accumulates in the form of inorganic Se (such as selenate SeO_4_^2−^ and selenite SeO_3_^2−^), as for elemental Se [Se(0)], it does not naturally occur in plants under normal conditions (Gupta and Gupta, 2017; Kieliszek, 2019; Cheng et al., 2024). The plant-derived Se is primary source of Se intake for humans. Se biofortification in plants is considered an effective strategy to addresses dietary Se deficiency in humans (Li et al., 2024). Since soil is the crucial source of this mineral for plants, its Se content and availability significantly affect the absorption and transformation of Se in plants, thus influencing human Se intake through the food chain (Kaur et al., 2022). However, due to the Se-deficient and low-availability soils across China, the Se content in plants is often insufficient, ultimately resulting in inadequate Se intake in humans (Dinh et al., 2019; Chen et al., 2020; Xie et al., 2021; Zhu et al., 2021). Therefore, enhancing the availability of Se in soil is a key approach to increase the Se concentration in plants.

Five different forms of Se primarily exists in the environment, including Se(-II), Se(0), Se(IV), and Se(VI) and organic Se (Lenz and Lens, 2009). In the Se(-II) state, Se typically occurs as hydrogen or ferrous selenide. These compounds have low solubility and are among the least stable forms of Se, resulting in relatively low mobility and availability (Wells and Stolz, 2020). Organic Se includes Se - containing amino acids methylselenides and trimethylselenonium ions (Zhao et al., 2024). The Se(0) has low mobility and is difficult for plants to absorb, but its chemical stability, makes it persist in soil over long periods (Winkel et al., 2015). Among these species, selenate exhibits the highest bioavailability, with organic Se, selenite, selenide, and elemental Se following in descending order (Zhao et al., 2024). Based on solubility and binding strength, the Se in the soil can be classified into five forms, including soluble Se (SOL-Se), exchangeable and carbonate-bound Se (EXC-Se), iron/manganese (Fe/Mn) oxide-bound Se (FMO-Se), organic matter-bound Se (OM-Se), and residual Se (RES-Se) (Wang et al., 2012). Among these, SOL-Se and EXC-Se are the most effective sources of Se for plant uptake (Chen et al., 2002; Ma et al., 2023). Since the availability of Se in the soil is influenced by its state and binding form, maintaining an adequate amount of highly mobile and easily absorbable Se species is essential in enhancing Se uptake in plants.

Microorganisms and environmental factors such as pH and redox potential jointly influence the states and binding forms of Se in the soil, thereby regulating its mobility and availability in soil (Winkel et al., 2015; Etteieb et al., 2020; Rosenfeld et al., 2020; Jiang et al., 2023; Moulick et al., 2024). Thus, identifying microorganisms exhibiting high and metabolic capacity for Se represents a critical approach to improve availability and utilization efficiency of soil Se resources. Currently, studies on the role of microorganisms in solubilizing Se to enhance its bioavailability are relatively limited and most research focuses on the mechanisms of Se reduction and methylation (Wells and Stolz, 2020; Ashengroph and Hosseini, 2021; Huang et al., 2021; Liu et al., 2023). Existing research indicates that Se-tolerant bacteria can promote the translocation and accumulation of Se from roots to shoots in crops (Kaur et al., 2022; Lin et al., 2022). To date, only a few bacterial strains such as *Bacillus*, *Thiobacillus ASN-1*, *Leptospirillum MNB-1*, *Agrobacterium*, *Dyella*, and *Rhodobacter* have been identified and reported to oxidize Se(-II) or Se(0) to Se(IV) and/or Se(VI) in the soil, and enhanced plant Se uptake (Zhu et al., 2021; Luo et al., 2022). However, the microbial mediation of Se transformation, as well as its subsequent availability through rhizosphere processes remain insufficiently underexploited.

Lettuce (*Lactuca sativa*) was selected as the experimental plant owing to its status as a globally important vegetable crop and its nutritional value for human health (Kim et al., 2016). In this study, we isolated *Bacillus amyloliquefaciens* strain G02 from seleniferous soil and determined its ability to dissolve Se(0) and Se mineral powder. With this bacterial strain G02, the objectives of this study were to (i) evaluate the potential roles of incubation with bacterial strain G02 on Se transformation and distribution in soil and its metabolomic mechanisms;(ii) assesse incubation with bacterial strain G02 impacts on the growth and Se enrichment in lettuce;(iii) reveal whether incubation with bacterial strain G02 can alter soil bacterial community, which could affect the Se uptake by lettuce. The results of this studyprovide valuable insight into understanding of microbially-mediated transformation of Se in soils and offer a novel perspective for developing targeted agronomic strategies for Se biofortification.

## 2 Materials and methods

### 2.1 Isolation and characterization of *Bacillus amyloliquefaciens* strain G02

Soil samples were collected from Qinzhou, Guangxi, China (N 21°57′36″, E 108°37′12″). Analytical methods outlined by Bao (2002) were employed to evaluate the chemical characteristics of the soil. The soil sample was mildly acidic (pH = 6.03) and comprised 28.33 g kg^−1^ of organic matter, 25.28 mg kg^−1^ of alkaline hydrolyzed nitrogen, 12.13 mg kg^−1^ of available phosphorus, and 71.20 mg kg^−1^ of available potassium. The total Se content measured was 1.32 mg kg^−1^. Approximately 10 g of soil was thoroughly mixed with 90 mL of sterile water using sterile glass beads and shaken at 150 rpm for 20 min at 28 °C and then serially diluted to 10^−1^, 10^−2^, and 10^−3^ concentrations. Exactly 0.1 mL of each dilution was cultured on the Luria-Bertani (LB) nutrient agar containing 1 mmol/L of selenite, incubated at 28 °C for 3 days and streaked on solid media five times to obtain pure and single colonies which were then preserved in glycerol or cultured in liquid media in a shaker until reaching the logarithmic phase. The isolated *B. amyloliquefaciens* strain G02 was stored at −80 °C to maintain their original characteristics and viability (Bhatt et al., 2023).

### 2.2 Screening, identification, and growth-promoting characteristics of the strain

Colonies at the logarithmic phase were inoculated on a sterile LB medium containing sodium selenite (Na₂SeO₃, 98%, produced by Tianjin Sitong Chemical Factory) solution at concentrations of 1, 10, 50, 100, 200, 300, 400, and 500 mM. The sodium selenite solutions were previously prepared using sterile water and filtered through a 0.22 μm membrane. The tolerance of *B. amyloliquefaciens* strain G02 to Na₂SeO₃ was determined by evaluating colony growth using both plate spreading and streaking methods over 48 h. Based on the observed tolerance range of 200–300 mM. The pure, single and tolerant colonies were then inoculated into 100 mL of LB broth and incubated at 180 rpm and 28 °C for 2 days to prepare the seed culture. Exactly 1% (v/v) of the seed culture was then inoculated on a 100 mL sterile LB broth containing either 0 or 2 mM Se and observed for growth at 0, 4, 8, 12, 20, 28, 36, 52, 76, and 100 h as described previously (Liao and Wang, 2022). Thereafter, 3 mL of each liquid culture was treated with Na_2_SeO_3_ and used to measure absorbance at 600 nm using a UV spectrophotometer (Cary 60, United States). Each treatment was conducted in triplicates. The growth curve of the strain was plotted based on the OD600 values measured in each treatment.

### 2.3 Morphological, physiological and biochemical characteristics of *Bacillus amyloliquefaciens* strain G02

The selected Se-tolerant bacterial strains were morphologically, physiologically and biochemically characterized as described previously (Zhu et al., 2019). Purified single colonies were streaked on plates and used to observe their growth morphology and photographed. Detailed methodologies for Gram staining identification, determination of growth temperature and pH range, 16S rRNA gene sequencing, assessment of phosphate-solubilizing efficiency, siderophore secretion, IAA (auxin) secretion, and ACC deaminase secretion by the strains are described in Supplementary Text S1.

### 2.4 Dynamic Se dissolution of Se-enriched mineral powder and elemental Se powder by the *Bacillus amyloliquefaciens* strain G02

The bacterial strain was incubated in 25 mL of 1/5 LB broth containing 0.025 g of Se powder [Se(0), black elemental Se powder, 98% purity] and 0.025 g of Se-rich mineral powder at 28 °C and 150 rpm for 5 days, with the broth without the strain as the control. The Se-rich mineral powder (SRMP) was composed of 0.13% Se (61.3% SiO₂, 15.2% Al₂O₃, 4.2% CaO, 3.4% K₂O, 2.2% MgO, 0.5% TiO₂, 0.2% Fe₂O₃ and 3.9% Na₂O) and was purchased from Hubei Jin Agricultural Technology Co., Ltd. The cultures were then centrifuged at 10,000 rpm for 10 min to obtain the supernatants, which were subsequently filtered through a 0.22 μm membrane. The pH of the filtrate was measured, and the Se(IV) concentration in the supernatants was determined using atomic fluorescence spectrometry (ICP-MS, NEXION 350×, PerkinElmer™ Life Science Incorporated, Waltham, MA, United States) as described (Zhu et al., 2021).

### 2.5 Metabolomic analysis of extracellular secretions from *Bacillus amyloliquefaciens* strain G02

The prepared strain G02 seed culture (10^8^ CFU/mL) was inoculated onto 25 mL of 1/5 LB broth containing 0.025 g of elemental Se powder [Se(0)] at a 1% inoculation ratio, and incubated with shaking at 28 °C and 150 rpm for 72 h, with the bacterial culture without Se(0) as the control (Shi et al., 2022). The experiment was repeated four times and was designated as SG (G02 + Se powder) or CG (G02 control). The cultures were then centrifuged at 10,000 rpm for 10 min to obtain the supernatants, which were filtered using a 0.22 μm sterile membrane, lyophilized in a vacuum freeze dryer (Alpha 1–2 LD plus, CHRIST Company) and then were sent to Shanghai Meiji Biomedical Technology Co., Ltd. for analysis. The detailed procedures for metabolite extraction and analysis were described in Supplementary Text S2.

Raw MS/MS data was processed using Progenesis QI software (Waters, Milford, MA, United States) for baseline filtering, peak identification, integration, retention time correction, and peak alignment. The resulting data matrix contained retention time, m/z, and peak intensity information. Substances with missing values >20% in each group were removed, and missing values were filled with the minimal value of each substance (per row). Total sum normalization was applied to eliminate system errors in various steps. Metabolic features with relative standard deviations >30% in QC samples were removed, and the remaining data were log10-transformed for subsequent analysis (Gu et al., 2021; Zhang et al., 2021). Metabolite identification was based on MS and MS/MS spectra with mass error tolerances set at ±10 ppm. Multivariate statistical analysis was performed using the ropls package (Version 1.6.2) in R. Principal component analysis (PCA) and orthogonal partial least squares-discriminant analysis (OPLS-DA) were conducted, with model stability assessed through 7-fold cross-validation. Metabolic pathway annotation and analysis were performed using the KEGG database.^1^ The study utilized both publicly accessible databases^2^ and a proprietary database of standard products developed by Shanghai Majorbio Bio-pharm Technology Co., Ltd. (Shanghai, China) (Yang et al., 2024). Differentially expressed metabolites (DEMs) with a variable importance in projection (VIP) value > 1 and a *p*-value < 0.05 were considered statistically significant (Meng et al., 2022). Based on the criteria of fold change (FC) > 1.2 or FC < 0.8, and *p* < 0.05 (Student’s t-test), a detailed analysis of significant DEMs was performed (Shi et al., 2024). All analyses were conducted on the Majorbio Cloud Platform^3^ (Ren et al., 2022).

### 2.6 Extraction of Se from soil by *Bacillus amyloliquefaciens* strain G02 incubation in a soil microcosm experiment

Exactly 6 × 10^8^ CFU/mL of the bacterial strains were suspended and homogenized within 20 g of soil, mixed with 0.5 mg/kg of each Se(0) and Na_2_SeO_3_ and then aged for 15 days in soil previously sterilized at 180 °C. The pH of the tested soil was 6.31, while the inoculated soil was incubated in sterile Petri dishes at 30 °C for 7 days. The treatment groups included Se(0) combined with strain G02 [Se(0)-G02], Se(IV) combined with strain G02 (SeIV-G02), a control group with Se(0) without inoculants [Se(0)-CK], and a control group with Se(IV) without inoculants (SeIV-CK). A soil sample was used to determine the pH, while air-dried samples were sieved using a 100-mesh sieve for further analysis. A continuous leaching method targeting SOL-Se, EXC-Se, FMO-Se, OM-Se, and RES-Se was used to extract Se forms from 1 g of soil (Wang et al., 2018).

### 2.7 Potted lettuce experimental design

Soil samples (20 kg) were collected from the 0–20 cm surface layer of farmland at Guangxi university. The soil was air-dried, finely ground, thoroughly mixed, and sieved through a 2 mm sieve to ensure homogeneity for further use. The homogenized soil exhibited the following physicochemical properties: pH 6.38, alkaline hydrolyzed nitrogen (6.98 mg/kg), available phosphorus (21.48 mg/kg), available potassium (210.2 mg/kg), and total Se (0.81 mg/kg). The potted lettuce experiments included the bacterial strain G02 inoculated treatment and an uninoculated treatment (CK) as the control. The experiment was conducted in triplicates, with three plants per replicate. Lettuce was planted in pots (height: 14 cm, diameter: 18 cm) filled with 2 kg of soil (Li et al., 2024; Guo et al., 2024). Base fertilizers were applied to each pot at the following dosages: NH₄Cl: 1.15 g/pot, KH₂PO₄: 0.19 g/pot, and KCl: 0.24 g/pot (all analytical grade; Yan, 2020). After germination, the lettuce seeds were first transplanted into a seedling substrate (Q/BVCG006-2017) for seedling growth. Uniformly developed seedlings were then selected and transplanted into the pots. The bacterial solution (10^8^ CFU/g) was applied twice using a sterile syringe: once at the time of transplantation and again a month later (Zhu et al., 2021). It was evenly distributed into the soil surrounding the lettuce roots. For the control group, sterile water was injected instead of the bacterial solution.

### 2.8 Determination of plant biomass and Se concentrations in samples

During soil sampling, a wooden stick was used to loosen the soil, while a small spoon was used to obtain rhizospheric soil samples. Fresh lettuce shoot and root samples were immediately placed in resealable bags, quickly frozen in liquid nitrogen, and later stored in a −40 °C freezer. The remaining plant samples were placed in envelopes, dried in an oven, ground into powder, and then stored at room temperature for further use. The soil samples were air-dried, homogenized, and sieved using 20, 50, and 100 mesh sieves before being stored at room temperature for further use. The fresh weight of the shoots and roots was measured using an analytical balance, and the average value per pot was calculated.

The concentration of Se in the plants was determined using the national standard GB 5009.93–2017, which employs a microwave digestion method (Chen et al., 2020). Exactly 0.2 g of the dried roots or shoots sample was placed in a digestion tube, alongside standard samples and blank controls, each containing 5 mL of concentrated HNO₃ and then digested using a microwave digester (CEM Mars6, United States). Afterwards, the acid was removed from the samples in a fumehood at 120 °C, and the liquid in the digestion tubes was reduced to 0.5 mL before cooling. The digestion tubes were then rinsed three times with ultrapure water and the resulting liquid was transferred to a 10 mL volumetric flask and made up to 10 mL. The samples were then filtered into centrifuge tubes, and Se concentrations in the plant were measured using an atomic fluorescence spectrometer (AFS, SA-20, Beijing, China). Bioavailable Se in soil was calculated as the sum of SOL-Se and EX-Se as described previously (Peng et al., 2016).

### 2.9 Determination of soil enzyme activity

The activities of sucrase (S-SC), catalase (S-CAT), acid phosphatase (S-ACP), and urease (S-UE) in the rhizospheric soil of lettuce were measured (Zhang et al., 2022). Enzyme activity assay kits were purchased from Solarbio Science & Technology Co., Ltd. (Beijing, China). The activities of the enzymes were determined according to the instructions provided in the manual, with calculations performed using the formulas specified by the manufacturer.

### 2.10 Effect of *Bacillus amyloliquefaciens* strain G02 incubation on bacterial diversity in rhizosphere soil

To determine the soil microbial diversity, the rhizospheric soil samples were collected from each treatment group in triplicates. Environmental microbial DNA was extracted using the Fast DNA™ SPIN Kit for Soil (MP Biomedicals), while its purity and concentration were assessed using 2% agarose gel electrophoresis and a Nanodrop 2000 spectrophotometer (Thermo Fisher Scientific, Wilmington, DE, United States). The 16S rRNA high-throughput sequencing analysis was carried out at the Shanghai Majorbio Bio-Pharm Technology Co., Ltd.^4^ The detailed methods for microbial DNA extraction from soil samples, 16S rRNA gene amplification, and BLAST-based strain identification are described in Supplementary Text S3.

The microbial communities in the rhizosphere soil of lettuce from the pot experiment were analyzed using 16S rRNA amplicon sequencing technology. The raw reads obtained were submitted to the NCBI SRA under the bacterial BioProject PRJNA1199144. All sample sequences were normalized to the minimum sequence depth across samples. Alpha diversity indices, including ACE, Chao1, Coverage, Shannon, Simpson, and Sobs, were calculated using the Mothur software^5^ (Douglas et al., 2020). A Venn diagram was generated to visualize the operational taxonomic units (OTUs) shared among different groups. Beta diversity was analyzed to assess the similarity of microbial community structures between samples. ANOSIM was used to test intergroup differences. In addition, partial least squares discriminant analysis (PLS-DA) was applied to examine group-level similarities and assess the significance of differences between groups. Linear discriminant analysis effect size (LEfSe) (Schloss et al., 2009)^6^ was used to identify bacterial taxa with significantly different abundances across groups at taxonomic levels ranging from phylum to genus (LDA > 3, *p* < 0.05).

### 2.11 Data analysis

The Se translocation and bioconcentration factors (BCFs) were calculated as described previously (Rahdarian et al., 2022) using the following formulas:


(1) $$\text{Translocation factor}\;(\mathrm{TF})=\frac{\mathrm{Se}\;\text{concentration in root}}{\mathrm{Se}\;\text{concentration in soil}}$$

(2) $$\text{Bioaccumulation factor}\;(\mathrm{BCF})=\frac{\mathrm{Se}\;\text{concentration in plant}}{\mathrm{Se}\;\text{concentration in soil}}$$

Data analysis was performed using SPSS 26 (IBM Corp., United States) and Excel 2021 (Microsoft Corp., United States). An independent sample Student’s *t*-test was used to compare results between the two groups, with statistical significance set at *p* < 0.05. Differences in means among multiple groups were analyzed using a one-way analysis of variance (ANOVA) and Duncan’s multiple range test, with a significance level of *p* < 0.05. Phylogenetic tree construction and data visualization were carried out using MEGA_64 (MEGA Limited, United States) and OriginPro 2024 (OriginLab Corp., United States), respectively.

## 3 Results and analysis

### 3.1 Characterization and qualitative analysis of the growth-promoting ability of *Bacillus amyloliquefaciens* strain G02

**Figure 1:**
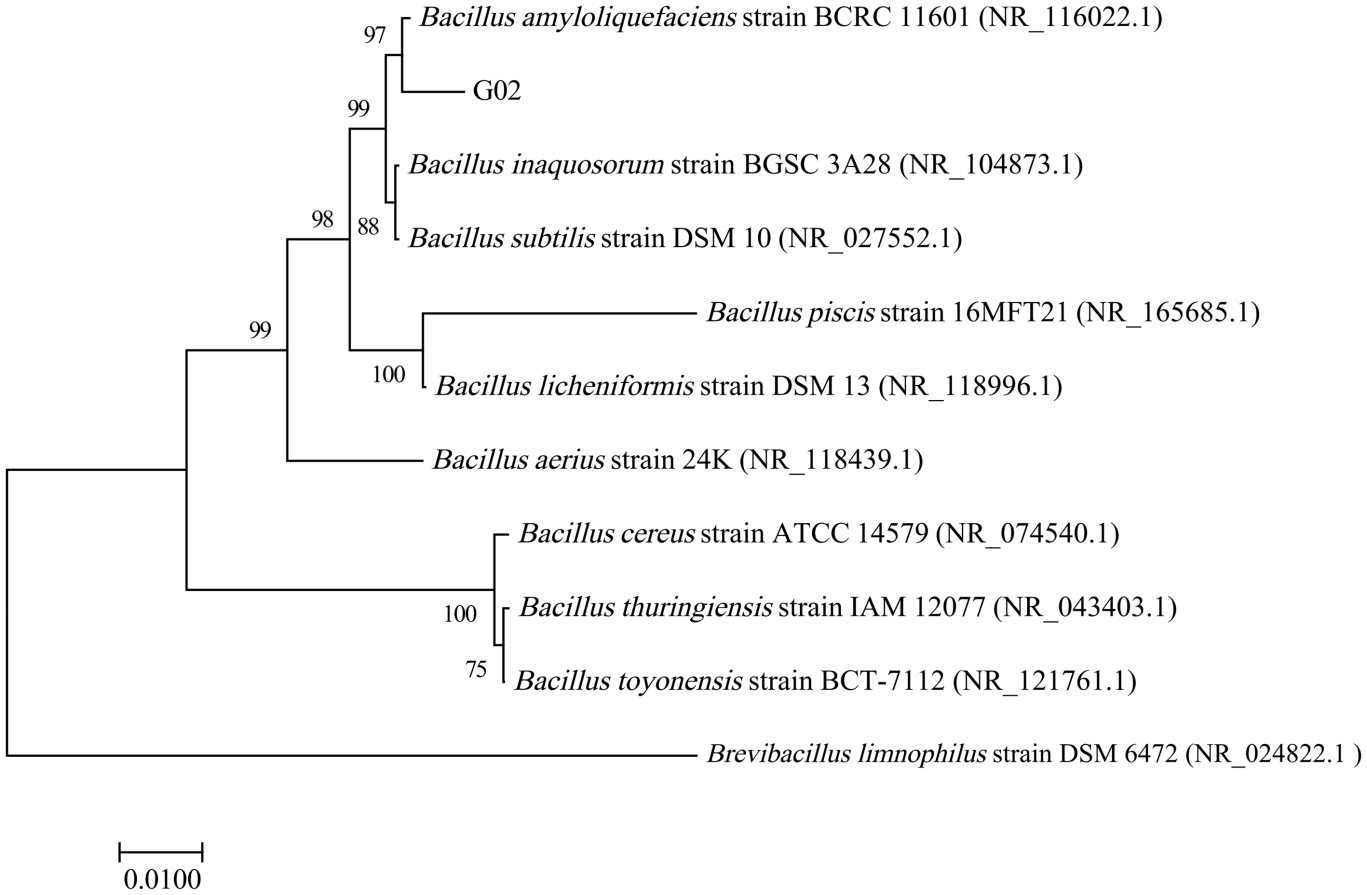
Phylogenetic tree based on 16S rRNA gene sequence showing the position of strain G02 and closely related species in the genus *Bacillus*.

The phylogenetic tree of the 16S rDNA (Figure 1) also clustered the strain G02 with *B. amyloliquefaciens* strain BCRC11601. The bootstrap values for the evolutionary tree exceeded 90, indicating a high level of reliability for the branch to which this strain belongs. Morphologically, the strain G02 bacterial strains were milky white with irregularly diffused round edges (Supplementary Figure S1a). Further microscopic analysis revealed that strain G02 is a gram-positive strain under a 100× oil lens (Supplementary Figure S1b). To evaluate the growth-promoting effects of the strain, key growth indicators were measured, revealing significantly higher PS and IAA production, while a lower relative expression of siderophores in the *B. amyloliquefaciens* G02-inoculated medium than in the non-inoculated medium. The *B. amyloliquefaciens* G02-inoculated medium also demonstrated the ability to secrete ACC deaminase (Table 1).

**Table 1:** Growth-promoting characteristic index.

Treatment	Phosphate solubilization (mg/L)	IAA production (mg/L)	Relative siderophore expression (As/Ar)	ACC deaminase secretion
Non-inoculated	7.26 ± 0.29b	−	1.02 ± 0.18a	−
G02	87.36 ± 0.65a	8.35 ± 0.26	0.49 ± 0.01b	+

The data is the average ± SD of the four repetitions. Different lowercase letters indicate that CK (Non-inoculated) is significantly different from the treatment group (G02) (*p* < 0.05).

### 3.2 Se solubilization ability of the *Bacillus amyloliquefaciens* strain G02 in Se powder and Se-rich mineral powder cultures

**Figure 2:**
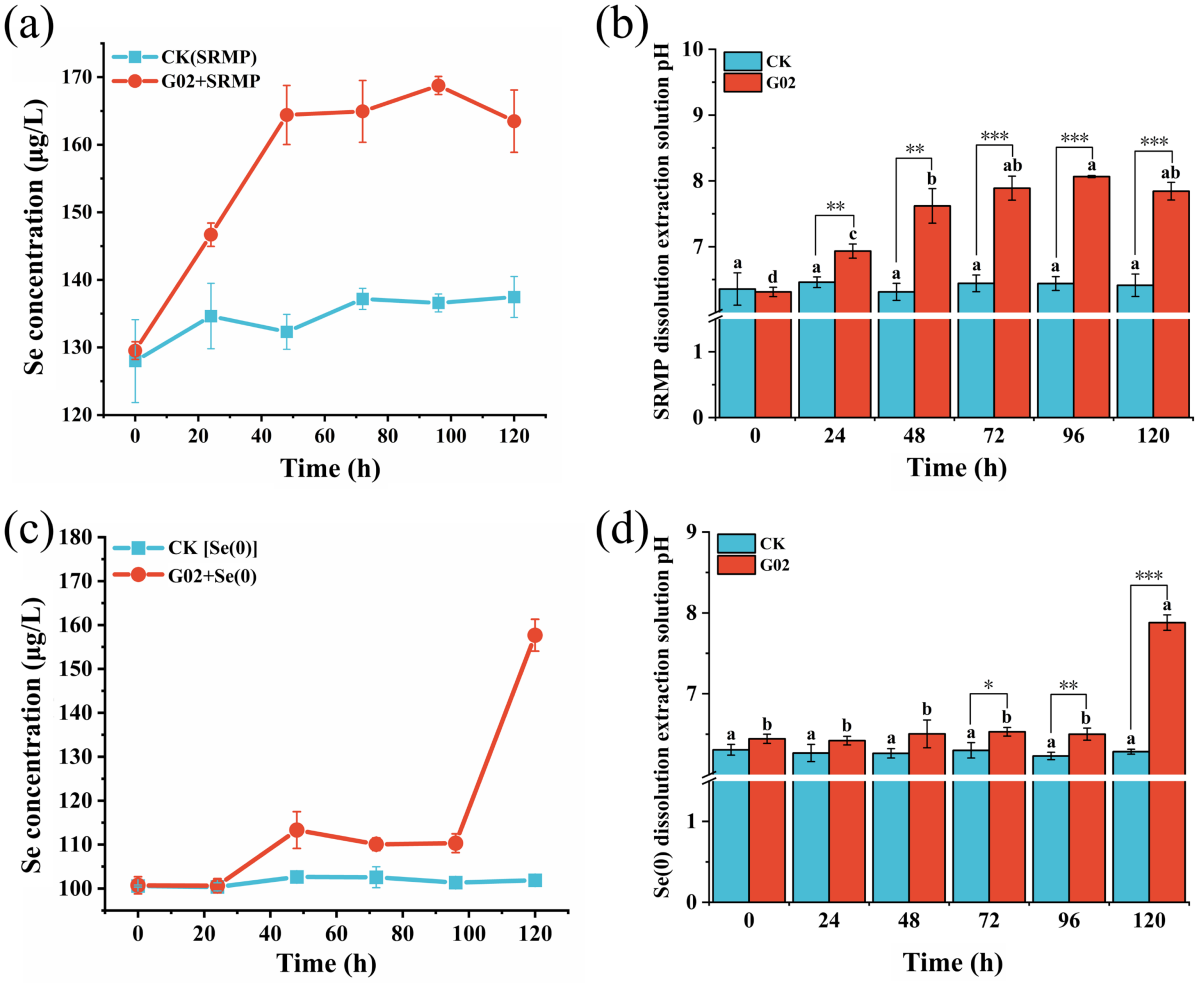
The dissolution of selenium by strain G02 and the pH changes. **(a)** Solubilization of Se-rich ore powder by strain G02; **(b)** The pH changes of the solution in the selenium-rich ore powder dissolution liquid; **(c)** Solubilization of elemental selenium powder by strain G02; **(d)** The pH changes of the solution in the elemental Se dissolution liquid. The data are mean ± SD of three replicates. Different lowercase letters indicate that there were significant differences in the same treatment (*p* < 0.05) and significant differences between different treatments at the same time are denoted as follows: (*p* ≤ 0.05, *), (*p* ≤ 0.01, **), and (*p* ≤ 0.001, ***).

The bacterial strain cultured in 2 mM Se exhibited a lag phase between 0 and 12 h, an exponential growth phase from 12 to 55 h, a stationary phase from 55 to 78 h, and an eventual decline phase after 78 h (Supplementary Figure S2). In the SRMP-containing medium, the concentration of Se in the strain of G02-inoculated treatment increased over time, with a significant rise from 0 to 48 h, and then stabilized between 48 and 120 h (Figure 2a). During the steady phase, the concentration of Se ranged from 164.4–168.76 μg/L, while the pH of the inoculated treatment began to diverge from the non-inoculated control group after the 24th hour (Figure 2b) reaching a pH of 8.07 at 96 h. This trend in pH change mirrored the variation in Se concentration, indicating that bacterial strain G02 possesses the ability to dissolve Se-rich mineral powder and thereby releasing Se. In the medium containing Se(0), the concentration of Se in the strain of G02-inoculated treatment remained relatively stable from 0 to 100 h, ranging from 110.32 to 113.33 μg/L, However, it rapidly increased after 100 h, reaching a peak of 157.68 μg/L at 120 h (Figure 2c). The pH of the G02 + Se(0) treatment began to show a significant difference after 72 h, reaching a maximum value of 7.88 at 120 h (Figure 2d). In the aquatic environment containing Se(0), the trend of pH changes was consistent with the increase in Se(IV) concentration following the inoculation of strain G02, with a significant rise in Se levels. These results indicated that inoculation of strain G02 can dissolve Se(0), while the pH changes may be related to extracellular metabolites produced during the metabolic processes in the strain.

### 3.3 Metabolomic analysis of the extracellular secretions of *Bacillus amyloliquefaciens* strain G02

**Figure 3:**
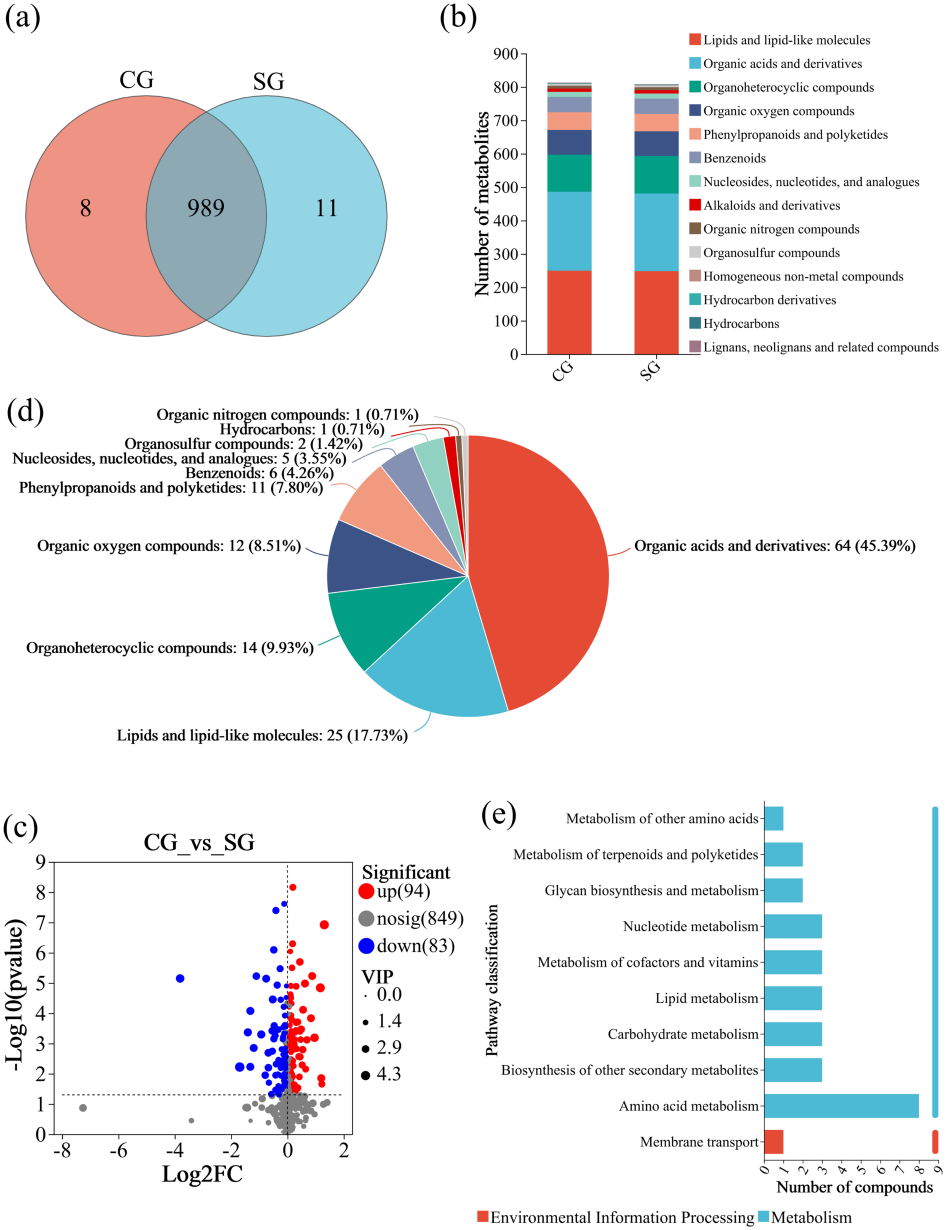
Metabolomic changes associated with Se(0) solubilization by strain G02. **(a)** The Venn diagram of extracellular metabolites of CG and SG; **(b)** Compound classification of the CG and SG groups; **(c)** Volcano diagram of differential metabolites between CG and SG; **(d)** Compound classification of CG_vs_SG; **(e)** KEGG pathway statistics of CG_vs_SG.

To explore the potential Se-dissolving and activating abilities of *B. amyloliquefaciens* strain G02, an untargeted metabolomic analysis of its extracellular secretions was conducted under the addition of Se(0). The PCA results (Supplementary Figure S3a) indicated classification differences between different treatment groups, while the samples within the same group exhibited good reproducibility. To further compare intergroup differences, orthogonal partial least squares discriminant analysis (OPLS-DA) scores (Supplementary Figure S3b) and model validation (Supplementary Figure S3c) were used to eliminate the possibility of random data correlations. The OPLS-DA results demonstrated that the metabolomic data of the samples were suitable for subsequent analysis. Exactly 989 metabolites were common between the CG and SG groups, eight metabolites were unique to the CG groups and 11 to the SG group (Figure 3a). These metabolites were annotated as lipids and lipid-like molecules, organic acids and derivatives, and organoheterocyclic compounds (Figure 3b). The volcano plot of the DEMs revealed 94 significantly upregulated and 83 significantly downregulated DEMs between in the CG_vs_SG comparison (Figure 3c).

To explore the functions of the DEMs in the CG_vs_SG comparison, compound classification analysis and KEGG pathway analysis were conducted. The compound classification showed that the main DEMs in the CG_vs_SG group were organic acids and derivatives comprising 45.39%, lipids and lipid-like molecules (17.73%), and organic heterocyclic compounds (9.93%), with other compounds accounting for relatively lower proportions (Figure 3d). The KEGG pathway analysis (Figure 3e) indicated that most DEMs (eight) were enriched in the amino acid metabolism. The other nine significantly enriched metabolic pathways included the biosynthesis of other secondary metabolites, carbohydrate metabolism, lipid metabolism, metabolism of cofactors and vitamins, and nucleotide metabolism, among others. These results collectively suggested that the addition of Se powder significantly altered the metabolite secretion patterns of bacterial strain G02. Based on the variable importance in projection (VIP) score (VIP > 1) and fold change (FC > 1.2 or FC < 0.8, *p* < 0.05), 16 DEMs out of organic acids and derivatives compounds showed marked changes in response to the two group treatments (Supplementary Table S1). It is noteworthy that metabolites (analogs) related to sulfur in formula, such as Histidinyl-Methionine, N-Formyl-L-methionine were significantly upregulated. These metabolites may be associated with the dissolution of Se(0) or the Se metabolism process.

### 3.4 Effects of *Bacillus amyloliquefaciens* strain G02 on the transformation of soil Se species

To explore the role of *B. amyloliquefaciens* strain G02 inoculation in transforming the Se binding states, a soil microcosm experiment of soil was conducted. The results showed that in the sterilized soil with Se(0) and Se(IV) as Se sources, an addition of strain G02 significantly affected the proportions of the five Se species in the soil (Supplementary Figure S4). Under the Se(0) treatment, the proportions of SOL-Se and EXC-Se increased by 1.7 and 9.6%, respectively, while the proportions of FMO-Se, OM-Se, and RES-Se decreased by 2.4, 5.5, and 3.4%, respectively. Under the Se(IV) treatment, SOL-Se and EXC-Se proportions increased by 2.9 and 8.5%, respectively, while FMO-Se, OM-Se, and RES-Se proportions decreased by 1.3, 9.1, and 0.9%, respectively. Additionally, soil pH significantly increased after the addition of strain G02 (Supplementary Table S2). These results indicated that Se species underwent significant changes following strain G02 inoculation, causing more Se to be present in water-soluble and exchangeable forms that are more readily absorbed by plants.

### 3.5 Effects of *Bacillus amyloliquefaciens* strain G02 inoculation on uptake and accumulation of Se in lettuce

**Figure 4:**
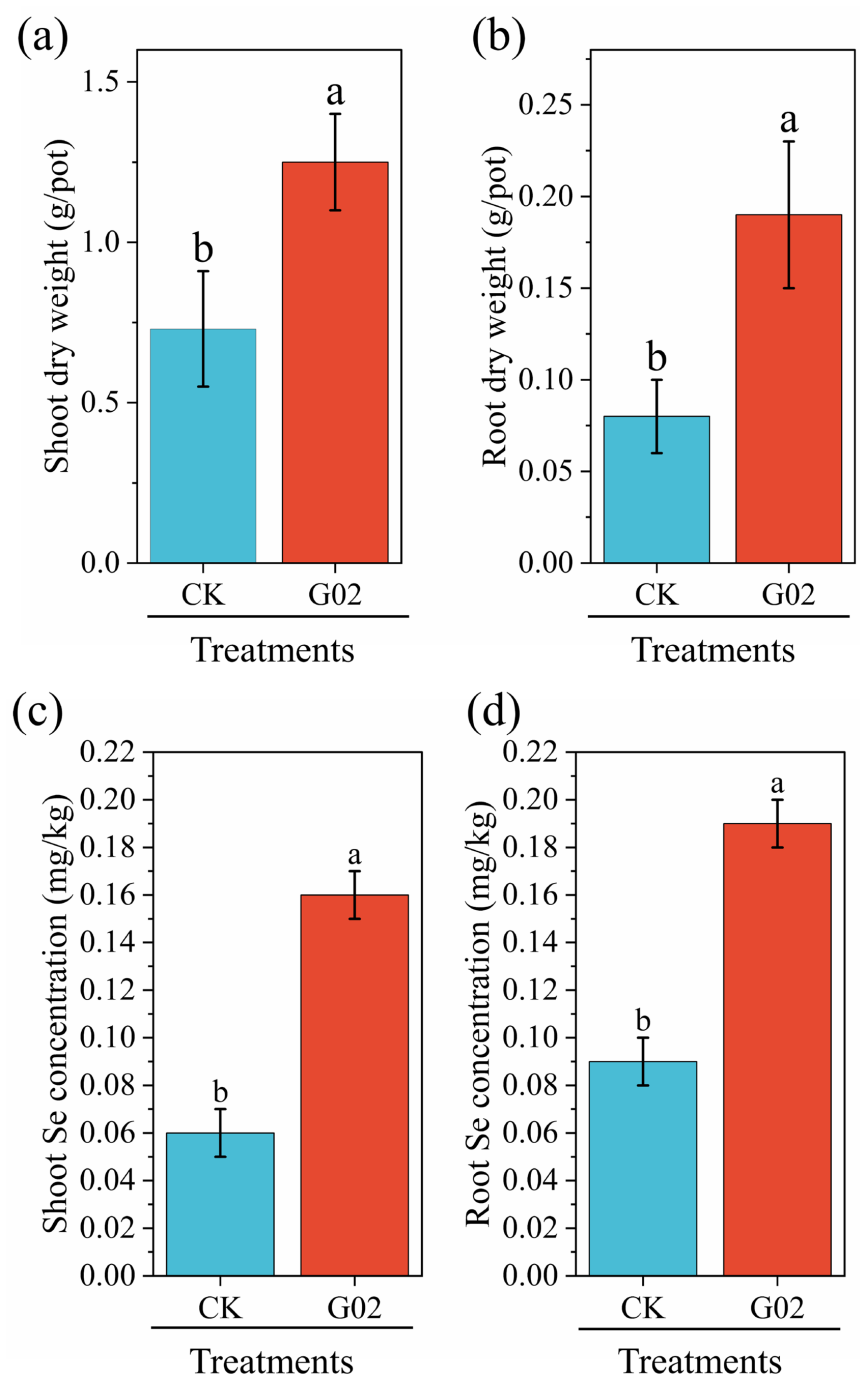
Biomass and Se concentration of lettuce. **(a)** Dry weight of shoot; **(b)** Dry weight of root; **(c)** Selenium concentration in shoot; **(d)** Selenium concentration in root. Data represent the mean ± standard deviation (SD) of three replicates. Different lowercase letters indicate significant differences between CK and treatment groups (*p* < 0.05).

Compared with the control group, the addition of strain G02 significantly (*p* < 0.05) increased the dry weight of the lettuce shoot (Figure 4a) and root (Figure 4b) increased by 60% and 1.4-fold, respectively. The inoculation of the strain G02 also significantly (*p* < 0.05) enhanced the concentration of Se in lettuce, with the concentrations in the roots increasing by 1.2-fold (Figure 4c) and those in the shoots increasing by 1.3-fold (Figure 4d). These results indicated that the inoculation of the strain G02 significantly boost the yield and has a Se-enriching effect on lettuce. Inoculation with strain G02 also significantly increased the soil-to-root transfer factor (TF_soil-root_) by 115.7% (Table 2). Additionally, strain G02 inoculation significantly enhanced the BCF of lettuce, increasing its Se enrichment capacity by 1.2-fold. Therefore, the strain G02 inoculation significantly improved the Se transfer capacity from soil to roots and markedly increased the BCF of Se in lettuce.

**Table 2:** Effects of inoculation with strain G02 on Se translocation and bioconcentration factor of lettuce.

Treatment	TF_soil-root_	BCF_soil-plant_
CK	0.115 ± 0.028b	0.081 ± 0.007b
G02	0.248 ± 0.034a	0.180 ± 0.026a

The data is the mean ± SD of three replicates. Different lowercase letters indicate that CK is significantly different from the treatment group (G02) (*p* < 0.05). TF, Translocation Factor, calculated using Equation (1); BCF, Bioaccumulation Factor, calculated using Equation (2).

### 3.6 Effects of *Bacillus amyloliquefaciens* G02 strain inoculation on available Se and enzyme activities in lettuce rhizospheric soil

In the potted experiment, strain of G02-inoculation significantly increased the concentration of available Se in the rhizospheric soil by 27.6% compared with the control groups (Supplementary Figure S5). The strain G02 inoculation also had a significant impact on the enzyme activities in the rhizospheric soil (Table 3), by significantly increasing the activities of catalase, sucrase, and acid phosphatase by 4.8, 73.0, and 16.9%, respectively (*p* < 0.05) compared with the controls. However, the activity of urease did not significantly change (*p* > 0.05). These results indicated that the strain G02 inoculation has varying effects on different enzymes, with a particularly pronounced impact on sucrase, which is related to carbohydrate metabolism.

**Table 3:** Changes of enzyme activity in rhizosphere soil of lettuce under different treatments.

Treatment	S-CAT activity	S-SC activity	S-ACP activity	S-UE activity
CK	96.33 ± 0.83b	0.63 ± 0.04b	26016.58 ± 723.14b	151.90 ± 1.32a
G02	100.96 ± 0.52a	1.09 ± 0.02a	30414.91 ± 447.92a	150.81 ± 1.26a

The data is the mean ± SD of three replicates. Different lowercase letters indicate that CK is significantly different from the treatment group (G02) (*p* < 0.05). S-SC, soil sucrase; S-CAT, soil catalase; S-ACP, soil acid phosphatase; S-UE, soil urease.

### 3.7 Effects of *Bacillus amyloliquefaciens* strain G02 inoculation on rhizosphere soil microbial diversity in lettuce

**Figure 5:**
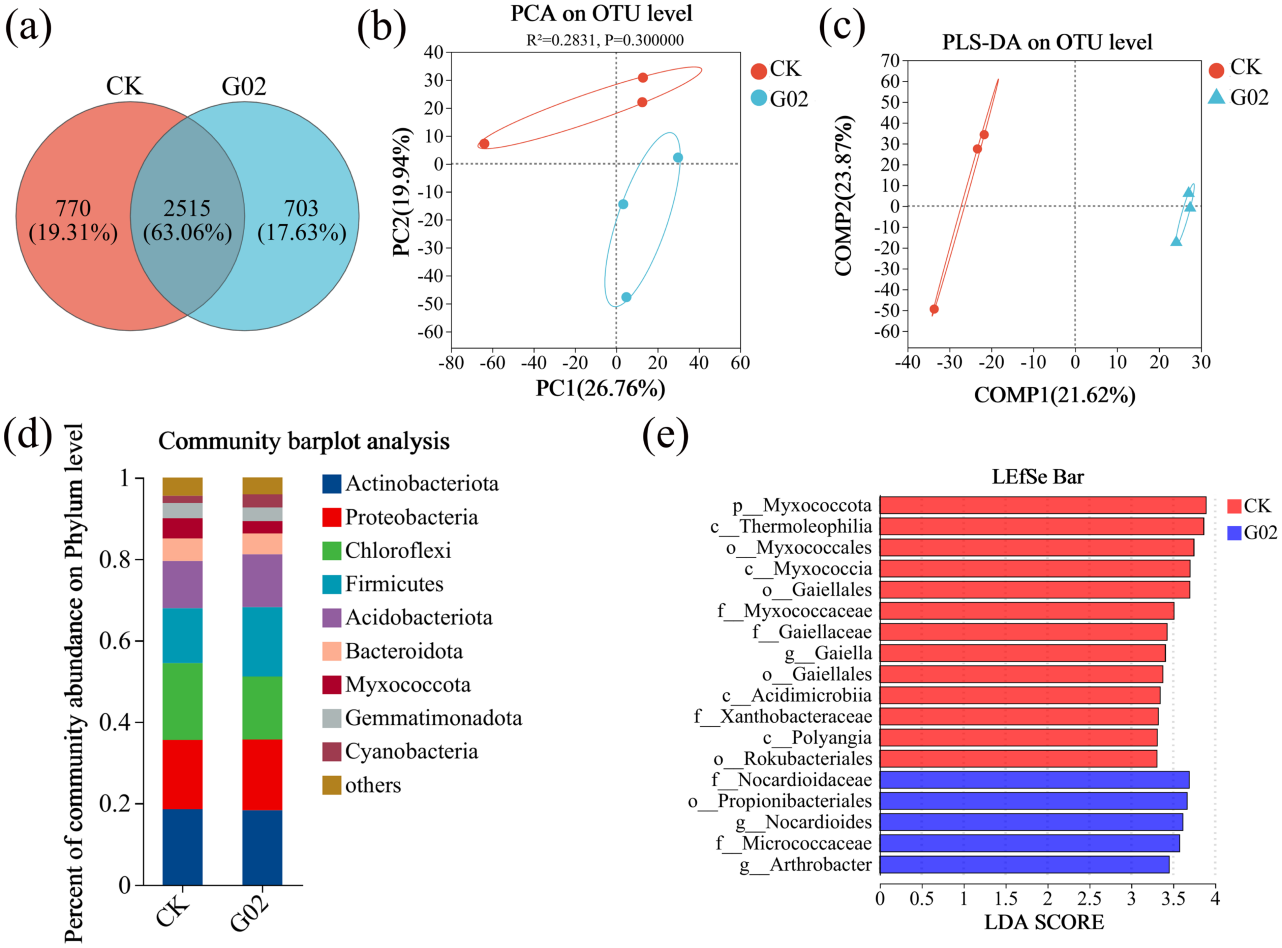
Analysis of microbial diversity in lettuce rhizosphere soil. **(a)** Venn diagram of species across different groups; **(b)** PCA analysis at the OTU level; **(c)** PLS-DA analysis of sample grouping at the OTU level; **(d)** Composition of rhizosphere soil microbiota at the phylum level; **(e)** LDA discriminant bar chart (showing only markers with LDA > 3.3). Microbial taxa with significant effects in the statistical grouping were identified using LDA (linear discriminant analysis). A higher LDA score indicate a greater contribution of species abundance to the observed differences.

The microbial composition in the non-G02-inoculated lettuce (CK) was 19.31 and 17.63% in strain of G02-inoculated lettuce (Figure 5a). Regarding community similarity, the PCA analysis results (Figure 5b), sample grouping analysis results (Figure 5c), and soil bacterial community diversity indices, including ACE, Chao1, Coverage, Shannon, Simpson, and Sobs (Supplementary Table S3), further confirmed that strain G02 inoculation caused a slight change in the overall composition of the microbial community, but did not reach a significant level (*p* > 0.05). The study shows that the phyla Actinobacteriota, Proteobacteria, Chloroflexi, Firmicutes, and Acidobacteriota are the most dominant species (Figure 5d). Compared to CK, the relative abundance of Actinobacteriota, Proteobacteria, and Bacteroidota showed minimal change of less than 1% after strain G02 inoculation. The relative abundance of Firmicutes, Acidobacteriota, and Cyanobacteria increased by 3.57, 1.37, and 1.52%, respectively, but decreased by 3.38 and 1.92% for Chloroflexi and Myxococcota, respectively. At the genus level (Supplementary Figure S6), the application of strain G02 increased the relative abundance of *Bacillus*, *Vicinamibacterales*, *Sphingomonas*, *Intrasporangium*, *Roseiflexaceae*, and *Nocardioides* by 26, 14, 6, 4, 14, and 22%, respectively. However, the relative abundance of *Flavisolibacter* decreased by 4% with the application of strain G02.

To gain a deeper understanding of the changes in rhizospheric microbial communities following the inoculation of strain G02, we conducted a multi-level species differential analysis (LEfSe) to identify key microbial taxa in the rhizosphere soil with significantly different relative abundances between treatments. The LEfSe multi-level species hierarchy tree (Supplementary Figure S7) showed significant differences in the abundance and taxonomic distribution of microbial communities under different treatment conditions. The results of the LDA discriminant bar chart (Figure 5e) indicated that in the control group, the unique taxa with notable differences included *p__Myxococcota*, *c__Thermoleophilia*, *o__Myxococcales*, and others. In the strain of G02-treated groups, the unique taxa such as *f__Nocardioidaceae*, *o__Propionibacteriales*, and *g__Nocardioides* contributed significantly to the differences between groups. These differences in the species may be related to specific metabolic products or environmental regulatory effects induced by strain G02 inoculation.

## 4 Discussion

### 4.1 Identification and growth-promoting characteristics of strain G02

*Bacillus amyloliquefacien*s is a typical plant growth-promoting rhizobacterium considered an important strain for the development of agricultural biofertilizers and biocontrol agents, and is also widely used to enhance plant tolerance to biotic and abiotic stresses (Dimopoulou et al., 2021). In this study, *B. amyloliquefaciens* strain G02 was screened and isolated from seleniferous soil. Based on 16S rRNA gene sequence analysis, it was identified as *B. amyloliquefaciens* BCRC11601 with 99.44% sequence similarity, since species with a similarity above 97% are classified within the same genus (Yadav et al., 2019).

In this study, the strain G02 demonstrated a mid-to-high-range phosphate-solubilizing capacity of 87.36 mg/L and significantly higher IAA production but a lower relative expression of siderophores in the strain of G02-inoculated lettuce than in the non-inoculated plants. The strain of G02-inoculated lettuce also demonstrated the ability to secrete ACC deaminase. Plants regulate their growth and development through small signaling molecules such as phytohormones and certain microorganisms can secrete plant hormones like IAA and gibberellins (Nascimento et al., 2019). As a plant hormone, IAA directly influences crop growth, particularly root elongation (Ma et al., 2022), the application of strain G02 in the rhizosphere of lettuce can modulate the hormone levels in the rhizosphere, thereby promoting growth. Furthermore, strain G02 possesses the ability to secrete siderophores and ACC deaminase. The ACC deaminase activity of strain can enhance plant stress tolerance (Hussain et al., 2020). Therefore, considering its Se dissolution capability, strain G02 can be used as a growth-promoting bacterial strain, thus making it a promising candidate for use as an organic fertilizer.

### 4.2 Metabolic regulation mechanism of strain G02 inoculation under elemental Se source

In Se-rich ore powder regions, Se occurs mainly in the Se(0) form and its dissolution is crucial in enhancing Se availability in soil. The process leads to the generation of oxyanionic forms of SeO₄^2−^ and SeO₃^2−^ with enhanced plant uptake efficacy (Deng et al., 2018). In this study, the strain G02 significantly increased the concentration of Se in the culture medium containing insoluble Se powder and Se ore powder, and also elevated the pH of the culture solution. Previous studies have indicated that the Se transformation by *B. amyloliquefaciens* strains is closely related to their reduction capacity (Ashengroph and Hosseini, 2021; Liu et al., 2023). In this study, the increase in Se concentration in the absence of other microorganisms suggests that the strain G02 can solubilize Se(0). Additionally, the increase in the pH may have also promoted the transformation of insoluble Se(0) into more soluble Se forms, thereby increasing Se mobility and availability to plants, as has been suggested (Staicu and Barton, 2017; Luo et al., 2022).

Bacteria acquire limiting nutrients, such as Fe and phosphorus by releasing protons or metabolites such as ligands or organic acids that affect pH (Ullman et al., 1996; Balland et al., 2010; Zhao et al., 2022). To investigate whether strain G02 metabolizes Se(0) as a source, we used a culture of strain G02 without Se(0) as a control. After adding Se(0), the metabolites in the inoculated strain G02 culture showed significant differences. According to the compound classification, the top three categories of DEMs were organic acids and derivatives (45.39%), lipids and lipid-like molecules (17.73%), and organic heterocyclic compounds (9.93%). The organic acids can compete with Se oxyanions for adsorption sites, thereby enhancing the availability of Se (Favorito et al., 2018). Therefore, the strain G02 may have altered the solubility of Se by secreting organic acids. On the other hand, sulfur (S) and Se share similar chemical properties, and Se may participate in or influence S metabolism and amino acid metabolism processes in microorganisms (Jiang et al., 2024). Previous studies have shown that the addition of sulfides and thiosulfates to biological cultures could significantly promote the dissolution of Se(0) (Dowdle and Oremland, 1998). Goff et al. (2019) isolated *Bacillus subtilis* JG17, which can dissolve Se(0) through extracellular reactions with active sulfur metabolites. Specifically, sulfide, sulfite, and thiosulfate react with Se(0) to form Se-sulfur aqueous species (SeS₂^−^), selenosulfate (SeSO₃^2−^), and selenothiosulfate (SeS₂O₃^2−^). These studies primarily focus on the role of inorganic sulfur compounds. In this study, we identified several S-containing metabolites (analogs) that were significantly upregulated during the experiment, such as N-Formyl-L-methionine and Histidinyl-Methionine. These sulfur-containing metabolites of the organic acid class may play a role in promoting the dissolution of Se(0). Our findings provide important clues for further exploring the role of S-containing metabolites in the dissolution of Se(0) ore powder.

### 4.3 Effects of strain G02 inoculation on Se speciation transformation in soil

In a soil microcosm experiment that excluded interference from other microorganisms, the inoculation of the strain G02 significantly altered the proportion of Se species in the soil. Soluble Se and exchangeable Se are highly bioavailable forms that are easily absorbed by plants (Hu et al., 2014; Ma et al., 2023). The inoculation of strain G02 increased the proportions of SOL-Se and EXC-Se while reduced the proportions of FMO-Se, organic sulfide-bound-Se, and RES-Se, thus increasing the concentration of bioavailable Se in soil. Additionally, the increase in soil pH promoted the conversion of poorly soluble Se into more soluble forms (Winkel et al., 2015). Therefore, strain G02 can play a role in activating Se in the soil.

### 4.4 Collaborative improvement of rhizosphere soil nutrients and enhanced Se accumulation in lettuce by strain G02 inoculation and microbial community

The abundance of microorganisms is typically represented by the ACE and Chao indices (Huang et al., 2017), while microbial diversity is estimated by the Shannon and Simpson indexes (Xun et al., 2018). In this study, microbial abundance and diversity slightly and insignificantly decreased after inoculation with strain G02. The soil microorganisms can increase Se availability in soil through metabolic activities such as extracellular phosphatase secretion (Jiang et al., 2023). In this study, the inoculation with strain G02 significantly enhanced the activities of soil catalase, sucrase, acid phosphatase, and urease. The activation of these enzymes influences soil nutrients and may play an important role in improving soil Se availability and lettuce biomass. Our results showed that inoculating strain G02 into the rhizospheric soil of lettuce significantly increased both root and shoot biomass and enhanced Se concentrations in various plant parts, promoting Se mobility and accumulation, and also increasing the concentration of available Se in the soil.

Inoculation with the strain G02 significantly enhanced Se absorption and accumulation in lettuce, primarily due to the successful colonization of G02 in the rhizosphere soil and its subsequent reshaping of the microbial community. The most direct evidence of this effect lies in the 26% increase in the relative abundance of G02, a *Bacillus* strain, following inoculation. *Bacillus*, recognized as an efficient plant growth-promoting genus, has been extensively documented for its ability to enhance Se uptake in plants (Lampis et al., 2009; Zhu et al., 2021; Luo et al., 2022). On this foundation, G02 colonization further triggered synergistic shifts within the microbial community. The abundances of several phyla closely associated with Se cycling and utilization, such as *Firmicutes*, *Acidobacteria*, and *Cyanobacteria*, were found to increase. These findings align with previous studies reporting their role in promoting Se absorption or transformation (Lei et al., 2011; Zhou et al., 2021; Devi et al., 2022). The enrichment of these beneficial microbial groups, coupled with the suppression of certain groups such as *Chloroflexi*, collectively indicates that the introduction of G02 optimized the rhizosphere microecology, creating an environment more conducive to soil Se activation and plant uptake (Feng et al., 2023). Therefore, we propose that the strain G02, through both its direct actions and its indirect regulation of microbial community structure, synergistically enhances Se uptake in lettuce.

## 5 Conclusion

The *B. amyloliquefaciens* strain G02 was selected for its significant plant growth-promoting ability. Metabolomic analysis revealed that the strain G02 secreted specific extracellular metabolites, including organic acids and sulfur-containing compounds. These substances may enhance the solubility of Se(0) and Se-rich mineral powders by promoting their dissolution. Additionally, inoculation with the strain G02 significantly increased the concentration of bioavailable selenium in the soil, thereby facilitating Se uptake and translocation in lettuce. Furthermore, G02 inoculation altered the rhizosphere microbial community structure, leading to an increase in the abundance of beneficial microorganisms, such as *Bacillus*, *Firmicutes*, and *Acidobacteria*. These microbes may work synergistically to activate and enrich selenium. These findings provide practical support for the development of efficient bio-selenium fertilizers. Future studies should further investigate the mechanism by which the strain G02 dissolves Se(0), while also evaluating its potential applications in different soil types and crop systems, as well as its stability and effectiveness under real-world agricultural conditions, to promote the widespread use of bio-selenium fertilizers.

## Data Availability

The datasets presented in this study are publicly available. This data can be found here: https://www.ncbi.nlm.nih.gov/sra, accession number PRJNA1199144.

## Footnotes

^1^ https://www.kegg.jp/kegg/pathway.html

^2^available at http://www.hmdb.ca and https://metlin.scripps.edu.

^3^ http://cloud.majorbio.com

^4^ http://www.majorbio.com

^5^ http://www.mothur.org/wiki/Calculators

^6^ http://huttenhower.sph.harvard.edu/LEfSe
