# Supplementary material for: Bacillus amyloliquefaciens G02 enhances selenium uptake in lettuce (Lactuca sativa) by improving soil selenium availability and rhizosphere microbiome regulation
Source: Front Microbiol. 2025 Sep 26;16:1656037. doi: 10.3389/fmicb.2025.1656037 (PMC12512348; doi:10.3389/fmicb.2025.1656037)
Supplement: Supplementary file 1 [file Supplementary_file_1.docx]

**Supplementary Text 1**

Gram Staining Identification. The Gram staining procedure was performed using a Gram staining kit (purchased from Solarbio Science & Technology Co., Ltd., Beijing). The staining steps included smear fixation, primary staining, mordant treatment, decolorization, and counterstaining, followed by microscopic examination. Deep purple staining indicated Gram-positive bacteria, while red staining indicated Gram-negative bacteria (Beveridge, 2001).

16S rRNA Gene Sequencing. Amplification of the 16S rRNA gene was conducted using the universal primers 27-F (5’-AGAGTTTGATCCTGGCTCAG-3’) and 1492-R (5’-GGTTACCTTGTTACGACTT-3’). PCR conditions were as follows: 95℃ for 5 minutes; 95℃ for 30 seconds, 56℃ for 30 seconds, and 72℃ for 1 minute 30 seconds, for 25 cycles; followed by 72℃ for 10 minutes and then cooling to 10℃. The PCR reaction mixture (20 μL total) consisted of 2.0 μL of 10× ExTaq buffer, 0.2 μL of 5u ExTaq, 1.6 μL of 2.5 mM dNTP Mix, 1 μL of each primer (5p Primer1 and Primer2), 0.5 μL of DNA, and 13.7 μL of ddH_2_O. The amplified samples were sent to Meiji Biomedical Technology Co., Ltd. (Shanghai) for genome extraction and sequencing. The raw sequences obtained through Sanger sequencing were subjected to quality control, and low-quality bases were removed to generate clean sequences. The clean sequences were compared with the NT database to identify the ten most similar species. Homology comparison of the 16S rDNA sequences was performed to construct a phylogenetic tree for strain classification and nomenclature.

The strains were inoculated onto PKO solid medium (inorganic phosphate bacteria medium, pH 7.0–7.5) and cultivated at 28℃ for 24 hours to measure phosphate solubilization (PS) efficiency, with solubilized phosphorus quantified using the molybdenum-antimony colorimetric method (Khourchi et al., 2022). For siderophore secretion, purified strains were grown in LB broth, and the supernatant was mixed with CAS detection reagent (pH 6.8) and measured at 680 nm (As), with the As/Ar ratio indicating siderophore production (Sultana et al., 2021). IAA secretion was assessed by adding Salkowski reagent to the supernatant, incubating in the dark for 30 minutes, and measuring absorbance at 535 nm, with IAA yield calculated using a standard curve (Gordon and Weber, 1951; Tiwari et al., 2016). ACC deaminase activity was evaluated by inoculating strains onto DF salt medium supplemented with 3 mM ACC, with colony growth indicating positive activity (Tiwari et al., 2016). Each experiment was repeated four times for accuracy.

**Supplementary Text 2**

All samples were placed in 2 mL centrifuge tubes containing 6 mm grinding beads and 400 µL of extraction solvent (methanol:water = 4:1, v/v) supplemented with 0.02 mg/mL internal standard (L-2-chlorophenylalanine). The samples were homogenized using a cryogenic tissue grinder at -10℃ and 50 Hz for 6 min, followed by low-temperature ultrasonic extraction at 5℃ and 40 kHz for 30 min. After standing at -20℃ for 30 min, the samples were centrifuged at 13,000 g and 4℃ for 15 min(Wen et al., 2022). The supernatant was transferred to injection vials with inserts for instrumental analysis. For quality control (QC) purposes, 20 µL aliquots from each sample supernatant were pooled to create QC samples. QC samples were prepared by combining equal volumes of extraction solvent from all samples. Each QC sample was processed and analyzed identically to the experimental samples. During instrumental analysis, one QC sample was injected every 5-15 experimental samples to monitor system stability and reproducibility.

Metabolite profiling was performed using an AB SCIEX UHPLC-Triple TOF 5600 system (Triple TOFTM5600, AB SCIEX, Foster City, CA, USA). Chromatographic separation was achieved on an ACQUITY UPLC HSS T3 column (100 mm ×2.1 mm i.d., 1.8 µm; Waters, Milford, USA) maintained at 40℃. The mobile phase consisted of (A) 95% water + 5% acetonitrile (0.1% formic acid) and (B) 47.5% acetonitrile + 47.5% isopropanol + 5% water (0.1% formic acid), with a flow rate of 0.40 mL/min. The injection volume was 10 μL. The gradient elution program was as follows: 0-2.5 min, 0-25% B; 3.5-9 min, 25-100% B; 9-13 min, 100% B; 13.0-13.1 min, 100-0% B; 13.1-16 min, 0% B for system equilibration. Mass spectrometric detection was performed using a Triple TOFTM5600 system equipped with an electrospray ionization (ESI) source. The scan range was set to 50-1000 m/z with the following parameters: ion source gas 1 (GS1) = 50 psi, ion source gas 2 (GS2) = 50 psi, curtain gas = 30 psi, ion source temperature = 550℃. The ionization voltage was set to +5000 V in positive mode and -4000 V in negative mode. Additional parameters included: interface heater on, declustering potential = 80 V, collision energy = 40±20 eV, and cycle time = 510 ms(Geng et al., 2021).

**Supplementary Text 3**

The microbial DNA was extracted from the soil samples using the E.Z.N.A. OMEGA-soil DNA Kit (Omega Bio-tek, Norcross, GA, USA) and amplified with 338F (5'-ACTCCTACGGGAGGCAGCA-3') and 806R (5'-GGACTACHVGGGTWTCTAAT-3') primers targeting the V3-V4 region of the bacterial 16S ribosomal RNA gene (Chen et al., 2018). The PCR was carried out in triplicates with a reaction mixture containing 4 μL of 5x FastPfu buffer, 2 μL of 2.5 mM dNTPs, 0.8 μL of each primer (5 μM), 0.4 μL of FastPfu polymerase, and 10 ng of template DNA. The amplified products were electrophoresed using 2% agarose gel, purified using an AxyPrep DNA Gel Extraction Kit (Axygen Biosciences, Union City, CA, USA), and quantified using QuantiFluor™-ST (Promega, USA). Each 16S rRNA gene sequence was classified using the RDP classifier (http://rdp.cme.msu.edu). The G02 strain was identified by amplifying and assembling its 16S rDNA sequence, followed by a BLAST analysis at the NCBI (https://www.ncbi.nlm.nih.gov/), indicating a 98.48% similarity between the strain G02 and *B. amyloliquefaciens* strain BCRC11601.


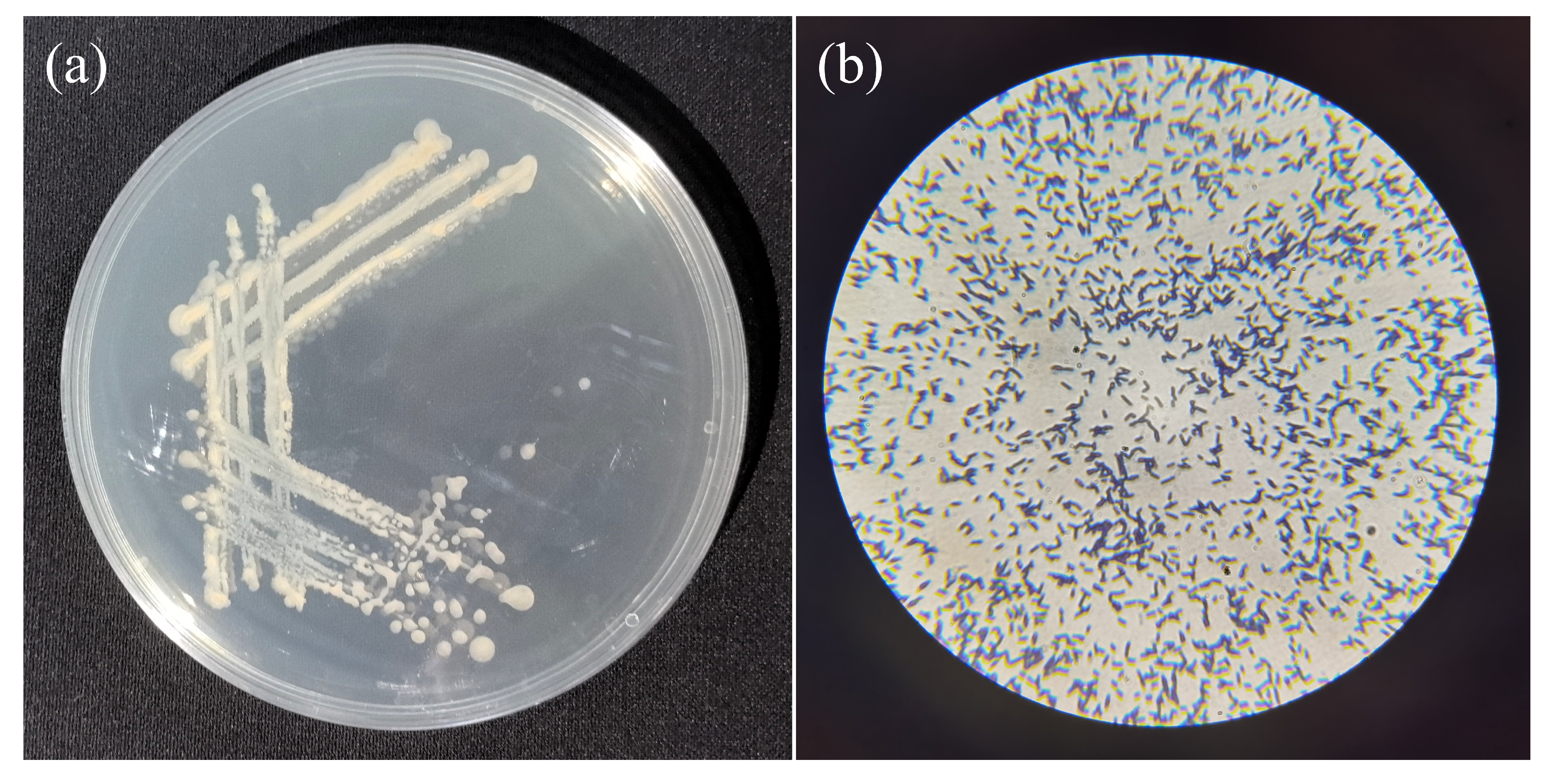


**Fig. S1.** Morphological characteristics of strain G02. (a) G02 strain streak plate morphology; (b) Strain Gram staining (100×) G02.


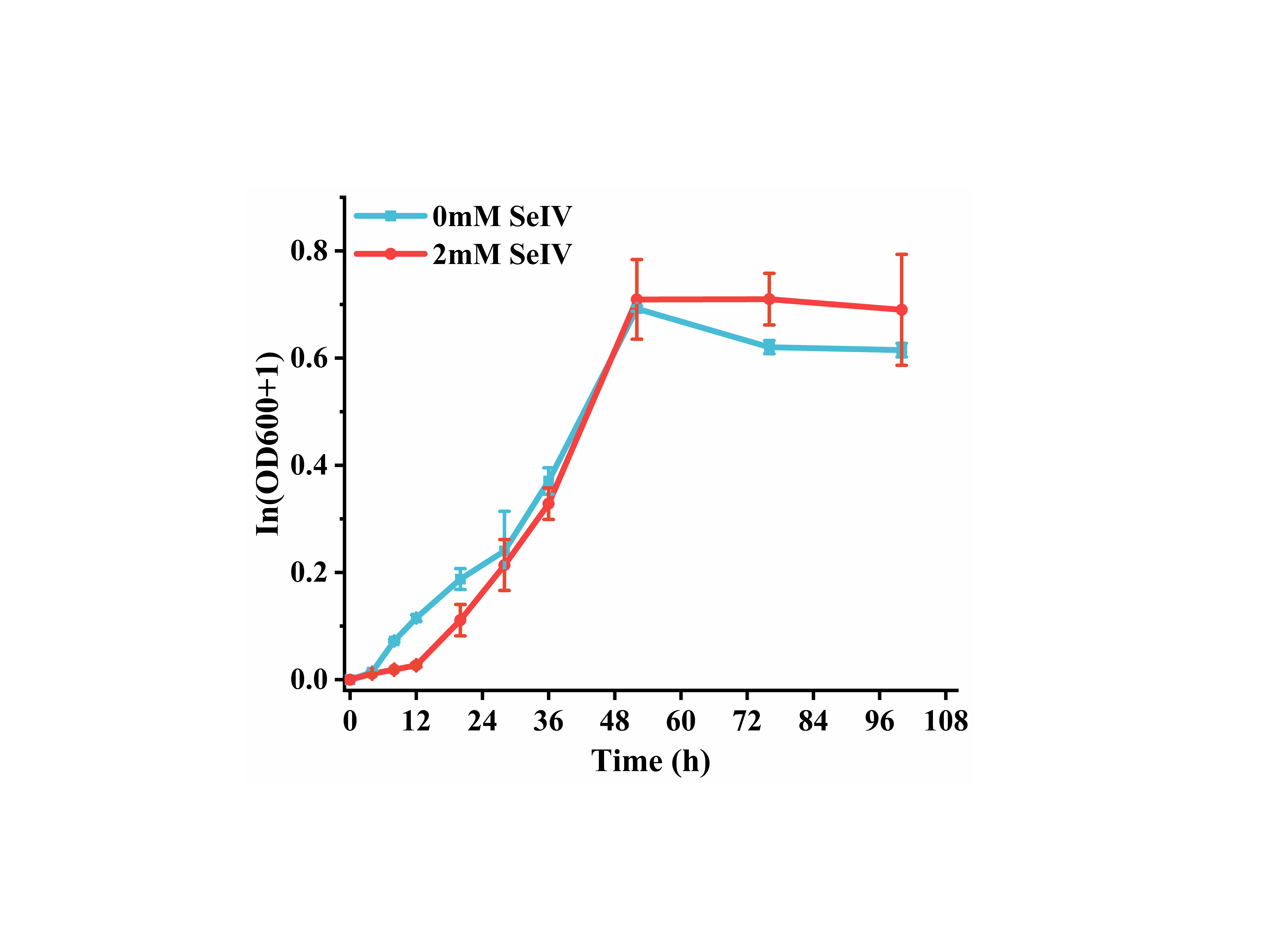


**Fig. S2.** The growth of strain G02 under 2mM Se(IV) treatment. Data represents the mean values of three replicates ± SD.





**Fig. S3**. (a) PCA analysis of samples in positive ion detection mode (left) and negative ion detection mode (right), (b) PLS-DA scores, and (c) PLS-DA model validation. **Note**: PLS-DA model validation. The x-axis represents the permutation retention level (the proportion consistent with the original model’s Y variable order, where a retention level of 1 indicates the original model's R2 and Q2 values), and the y-axis shows the R2 (blue circles) and Q2 (red triangles) values obtained from the permutation test, with the two dashed lines indicating the regression lines for R2 and Q2. The permutation test was conducted on both the experimental and control groups together. The permutation model involved randomly shuffling the group labels (Y variable) of the experimental and control groups. The x-axis's permutation retention level represents the proportion consistent with the original model’s Y variable order, and a retention level of 1 represents the R2 and Q2 values of the original OPLS-DA/PLS-DA model. The number of random permutations was set to 200. The evaluation criterion for the permutation test is to observe the intercept of the Q2 regression line with the Y-axis; an intercept of less than 0.05 indicates that the model is robust and reliable, without overfitting.


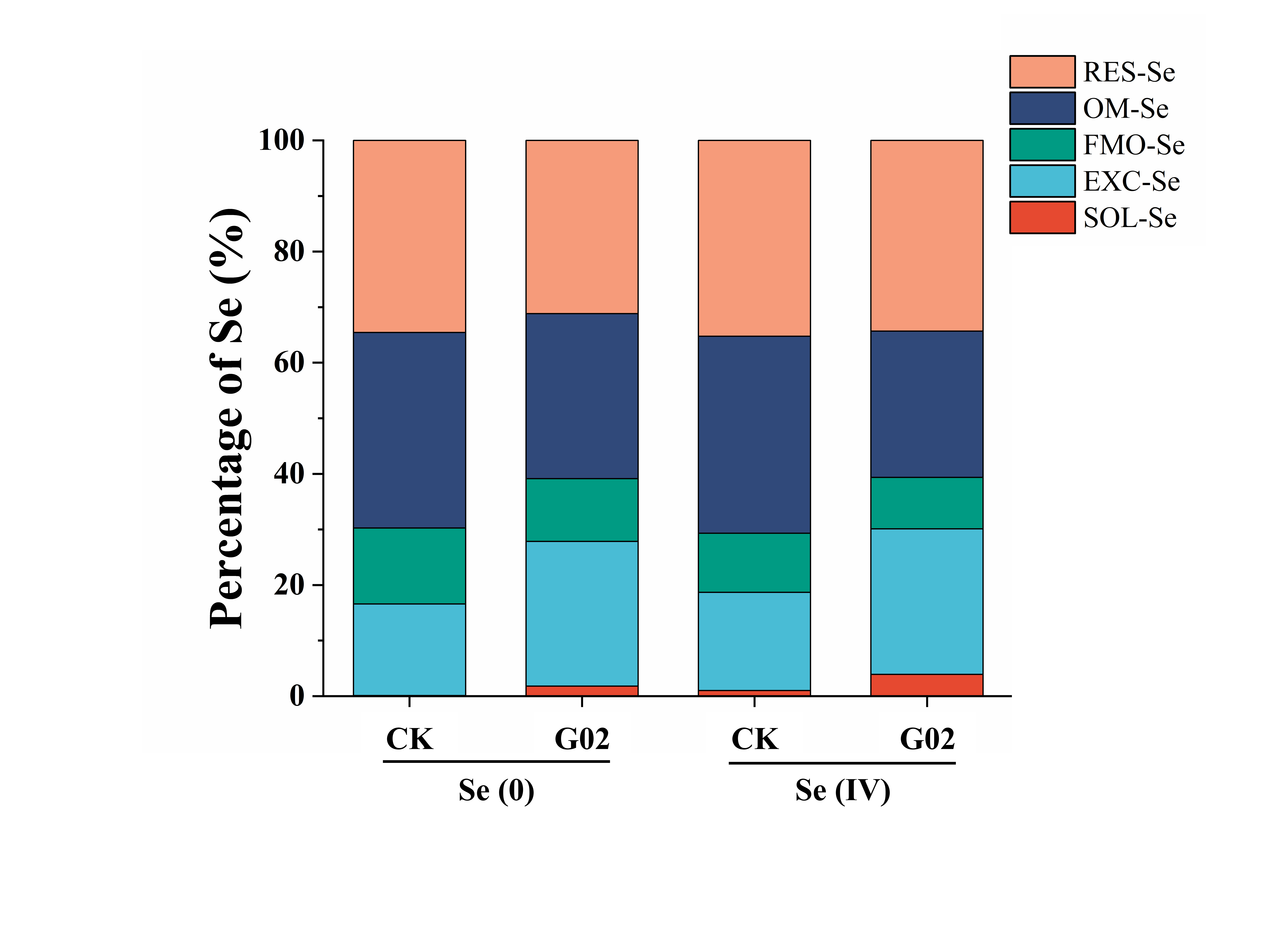


**Fig. S4.** Effects of exogenous application of G02 on soil Se forms. Note : Soluble Se ( SOL-Se ), exchangeable-carbonate-bound Se ( EXC-Se ), iron-manganese oxide-bound Se ( FMO-Se ), organic sulfide-bound Se and elemental Se ( OM-Se ), residual Se ( RES-Se ). Note : The data are the average of three replicates.


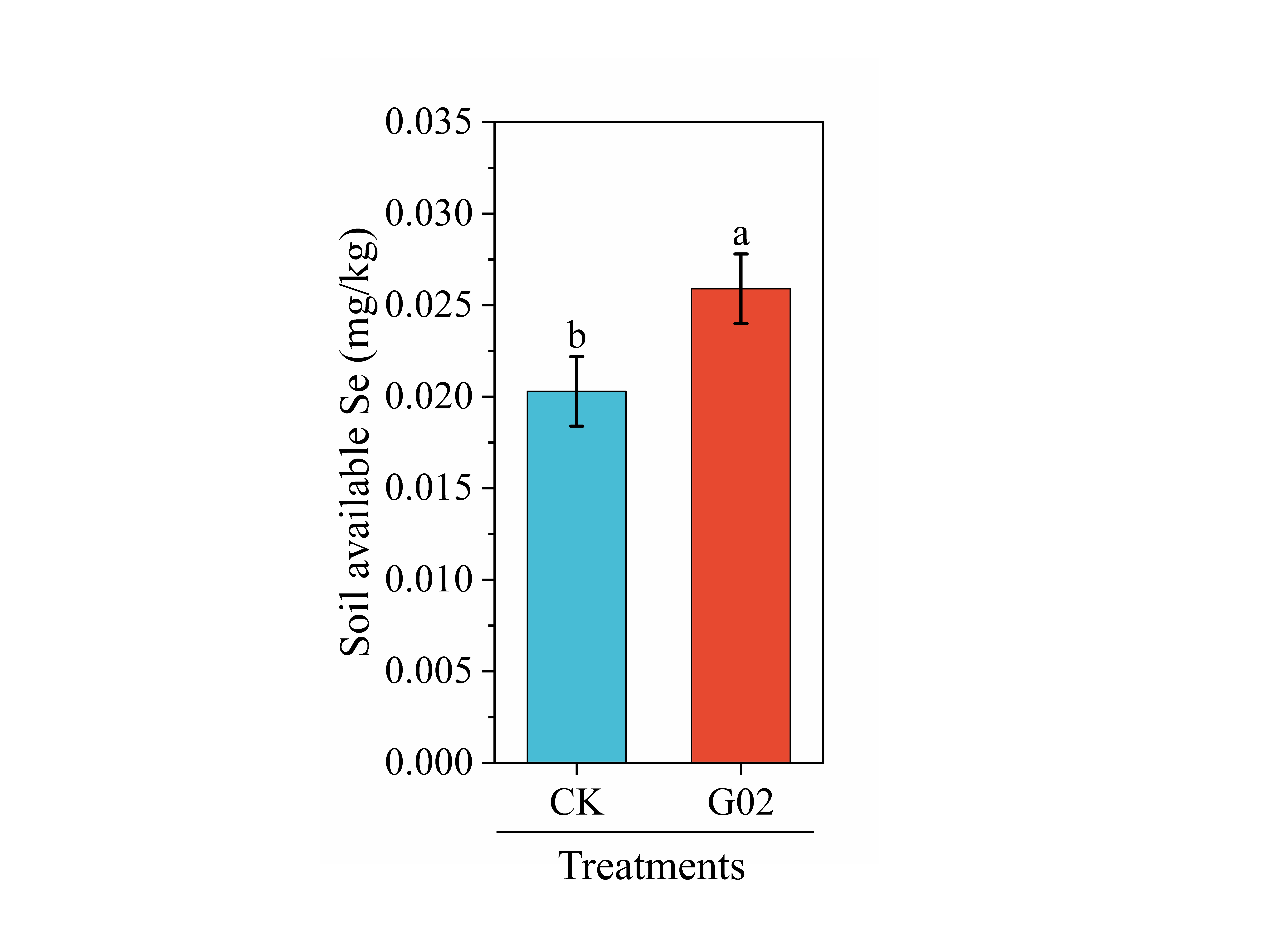


**Fig. S5**. Pot soil available Se concentration in lettuce. Note : The data is the mean ± SD of three replicates. Different lowercase letters indicate that CK is significantly different from the treatment group (G02)( *p* < 0.05 ).


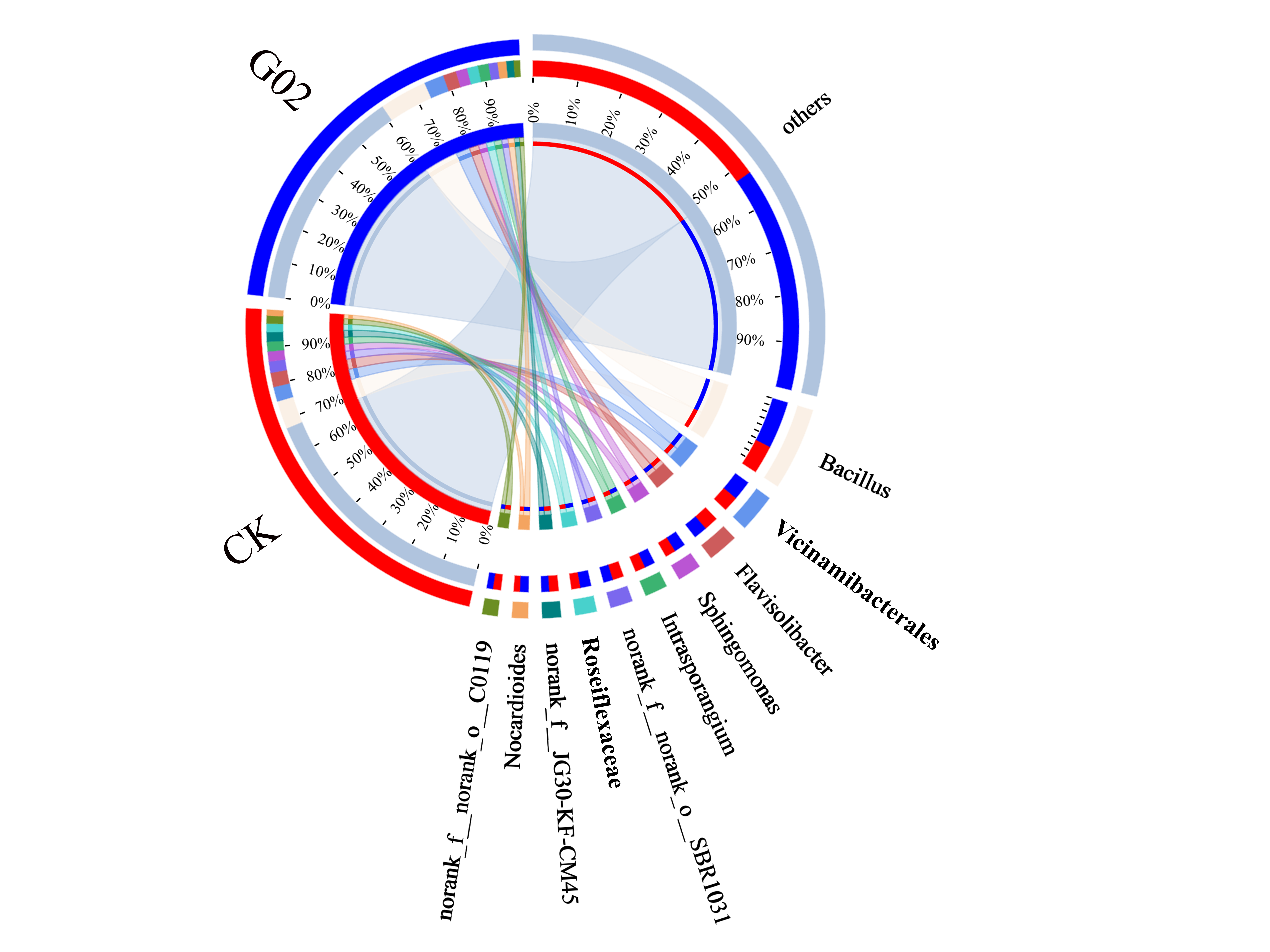


**Fig. S6**. Relationship between Samples and Species (Genus Level). **Note**: The left semi-circle represents the species composition within each sample. The color of the outer ribbons indicates the group from which each sample originates, while the color of the inner ribbons indicates the species, with the length representing the relative abundance of the species in the corresponding sample. The right semi-circle illustrates the distribution proportion of each species across different samples at this taxonomic level. The outer ribbons represent the species, while the inner ribbon colors represent different groups, with the length indicating the proportion of a given species in each sample. Taxa labeled with “norank_” typically represent microorganisms that have not been fully classified.


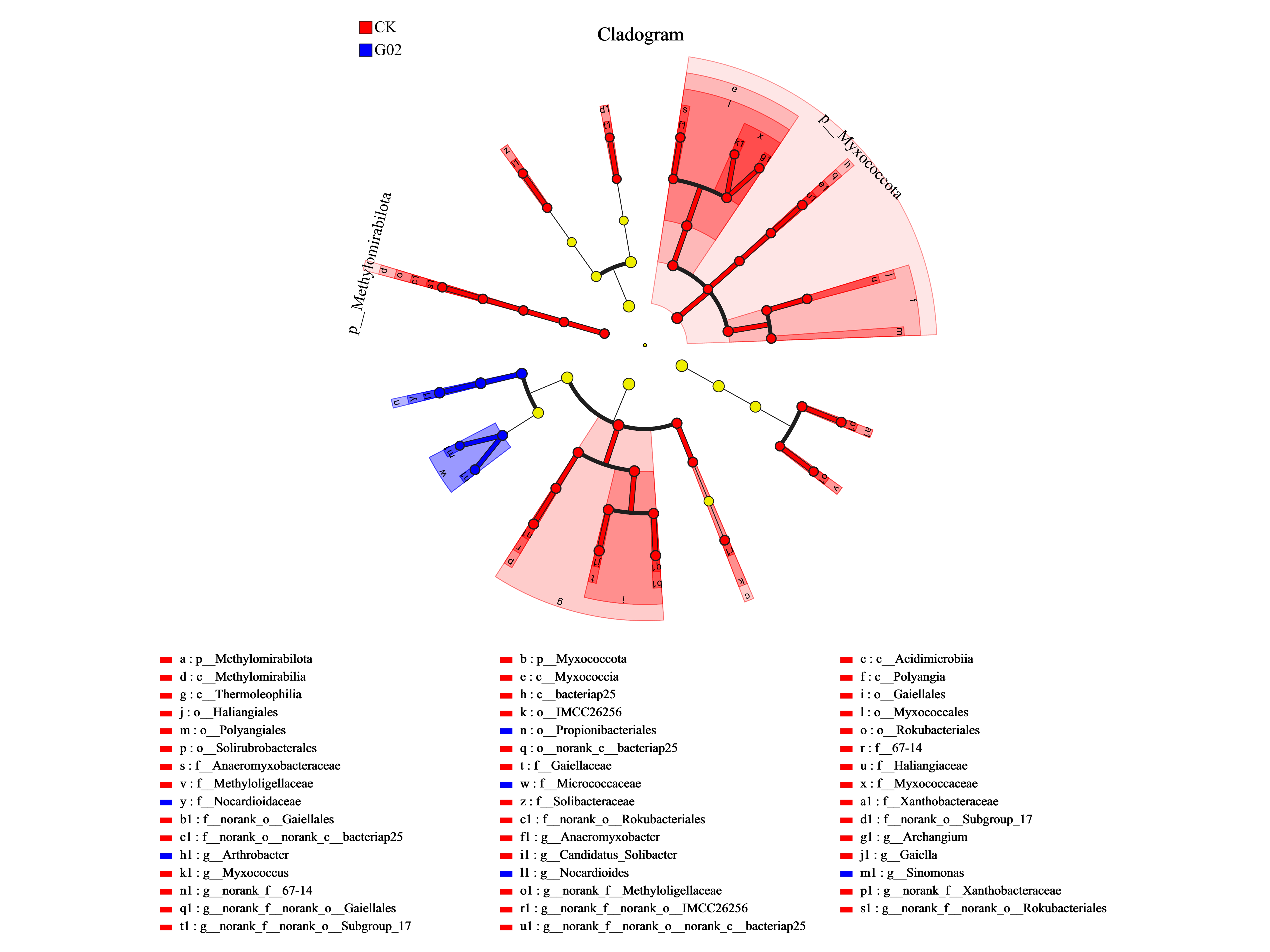


**Fig. S7**. Biomarkers in Different Treatments Based on LEfSe Analysis. **Note**: The concentric rings from the innermost to the outermost correspond to the taxonomic ranks of phylum and genus. In the phylogenetic tree, the colored sections highlight the taxonomic units that exhibit significantly higher relative abundance in the Kruskal-Wallis test (*p* < 0.05) and have a logarithmic LDA score greater than 3. These highlighted areas indicate species that are considered biomarkers under the different treatments. Taxa labeled with “norank_” typically represent microorganisms that have not been fully classified.

**Table S1 CG_vs_SG Differentially expressed metabolites statistics**

| Metabolite | VIP | FC(SG/CG) | Regulate | Formula |
| --- | --- | --- | --- | --- |
| Phenylalanylproline | 3.0 | 0.4 | down | C₁₄H₁₈N₂O₃ |
| Trimethylammonioacetate | 2.7 | 0.5 | down | C₅H₁₂NO₂⁺ |
| Leu Ala Ile | 3.1 | 0.5 | down | C₁₅H₂₉N₃O₄ |
| Capryloylglycine | 2.6 | 0.7 | down | C₁₀H₁₉NO₃ |
| Glutamylhistidine | 2.1 | 0.7 | down | C₁₁H₁₆N₄O₅ |
| L-cis-4-(Hydroxymethyl)-2-pyrrolidinecarboxylic acid | 2.4 | 0.8 | down | C₆H₁₁NO₃ |
| Guanidylic acid (guanosine monophosphate) | 1.6 | 0.8 | down | C₁₀H₁₄N₅O₈P |
| N8-Acetylspermidine | 1.7 | 0.8 | down | C₉H₂₁N₃O |
| Histidinyl-Methionine | 1.6 | 1.2 | up | C₁₁H₁₈N₄O₃S |
| Glycyl-Tryptophan | 1.7 | 1.2 | up | C₁₃H₁₅N₃O₃ |
| N(6)-(Octanoyl)lysine | 2.0 | 1.2 | up | C₁₄H₂₈N₂O₃ |
| Norophthalmic acid | 2.1 | 1.2 | up | C₁₀H₁₇N₃O₆ |
| N-Formyl-L-methionine | 1.7 | 1.3 | up | C₆H₁₁NO₃S |
| Prolyl-Tryptophan | 2.7 | 1.4 | up | C₁₆H₁₉N₃O₃ |
| Gamma-Glutamylphenylalanine | 3.0 | 1.5 | up | C₁₄H₁₈N₂O₅ |
| D-4'-Phosphopantothenate | 3.0 | 1.8 | up | C₉H₁₈NO₈P |

****Table S2**** Soil pH value of micro-culture

| Treatments | pH |
| --- | --- |
| Se0-CK | 6.35±0.01c |
| Se0-G02 | 6.63±0.04a |
| SeIV-CK | 6.45±0.02b |
| SeIV-G02 | 6.67±0.04a |

**Note : The data is the mean ± SD of four replicates. Different lowercase letters indicate that CK is significantly different from the treatment group (** *p* **< 0.05 ).**

**Table S3 Alpha diversity index table**

| Type | CK | G02 |
| --- | --- | --- |
| ace | 2669.9014 ± 67.2403a | 2589.1179 ± 67.8015a |
| chao | 2601.4056 ± 51.5877a | 2522.6181 ± 66.0423a |
| coverage | 0.9819 ± 0.0003a | 0.9817 ± 0.0006a |
| shannon | 6.2936 ± 0.1312a | 5.9886 ± 0.0953a |
| simpson | 0.0073 ± 0.0018a | 0.0131 ± 0.004a |
| sobs | 2206.3333 ± 101.988a | 2110.6667 ± 84.626a |

Note : The data is the mean ± SD of three replicates. Different lowercase letters indicate that CK is significantly different from the treatment group ( *p* < 0.05 ).

**Reference**

**Beveridge TJ (2001)** Use of the Gram stain in microbiology. Biotech Histochem 76(3):111-118. [https://doi.org/10.1080/bih.76.3.111.118](https://doi.org/10.1080/bih.76.3.111.118)

**Sultana S, Alam S, Karim MM (2021)** Screening of siderophore-producing salt-tolerant rhizobacteria suitable for supporting plant growth in saline soils with iron limitation. J Agric Food Res 4:100150. [https://doi.org/10.1016/j.jafr.2021.100150](https://doi.org/10.1016/j.jafr.2021.100150)

**Khourchi S, Elhaissoufi W, Loum M, Ibnyasser A, Haddine M, Ghani R, Barakat A, Zeroual Y, Rchiad Z, Delaplace P, Bargaz A (2022)** Phosphate solubilizing bacteria can significantly contribute to enhance P availability from polyphosphates and their use efficiency in wheat. Microbiol Res 262:127094. [https://doi.org/10.1016/j.micres.2022.127094](https://doi.org/10.1016/j.micres.2022.127094)

**Chen S, Zhou Y, Chen Y, Gu J (2018)** Fastp: an ultra-fast all-in-one FASTQ preprocessor. Bioinformatics 34(17):884-890. [https://doi.org/10.1093/bioinformatics/bty560](https://doi.org/10.1093/bioinformatics/bty560)

Geng F, Xie Y, Wang Y, Wang J (2021) Depolymerization of chicken egg yolk granules induced by high-intensity ultrasound. *Food Chem* 354：129580. https://coi.org/10.1016/j.foodchem.2021.129580.

Wen X, Geng F, Xu Y, Li X, Liu D, Liu Z, Luo Z, Wang J (2022) Quantitative transcriptomic and metabolomic analyses reveal the changes in tricholoma matsutake fruiting bodies during cold storage. *Food Chem* 381: 132292. https://coi.org/10.1016/j.foodchem.2022.132292.

**Gordon SA, Weber RP (1951)** Colorimetric estimation of indoleacetic acid. Plant Physiol 26(1):192–195. [https://doi.org/10.1104/pp.26.1.192](https://doi.org/10.1104/pp.26.1.192)

**Tiwari S, Sarangi BK, Thul ST (2016)** Identification of arsenic resistant endophytic bacteria from Pteris vittata roots and characterization for arsenic remediation application. J Environ Manage 180:359–365. [https://doi.org/10.1016/j.jenvman.2016.05.029](https://doi.org/10.1016/j.jenvman.2016.05.029)
